# Emerging Role of Neuron-Glia in Neurological Disorders: At a Glance

**DOI:** 10.1155/2022/3201644

**Published:** 2022-08-22

**Authors:** Md. Mominur Rahman, Md. Rezaul Islam, Md. Yamin, Md. Mohaimenul Islam, Md. Taslim Sarker, Atkia Farzana Khan Meem, Aklima Akter, Talha Bin Emran, Simona Cavalu, Rohit Sharma

**Affiliations:** ^1^Department of Pharmacy, Faculty of Allied Health Sciences, Daffodil International University, Dhaka, Bangladesh; ^2^Department of Pharmacy, BGC Trust University Bangladesh, Chittagong 4381, Bangladesh; ^3^Faculty of Medicine and Pharmacy, University of Oradea, P-ta 1 Decembrie 10, 410087 Oradea, Romania; ^4^Department of Rasa Shastra and Bhaishajya Kalpana, Faculty of Ayurveda, Institute of Medical Sciences, Banaras Hindu University, Varanasi, 221005 Uttar Pradesh, India

## Abstract

Based on the diverse physiological influence, the impact of glial cells has become much more evident on neurological illnesses, resulting in the origins of many diseases appearing to be more convoluted than previously happened. Since neurological disorders are often random and unknown, hence the construction of animal models is difficult to build, representing a small fraction of people with a gene mutation. As a result, an immediate necessity is grown to work within in vitro techniques for examining these illnesses. As the scientific community recognizes cell-autonomous contributions to a variety of central nervous system illnesses, therapeutic techniques involving stem cells for treating neurological diseases are gaining traction. The use of stem cells derived from a variety of sources is increasingly being used to replace both neuronal and glial tissue. The brain's energy demands necessitate the reliance of neurons on glial cells in order for it to function properly. Furthermore, glial cells have diverse functions in terms of regulating their own metabolic activities, as well as collaborating with neurons via secreted signaling or guidance molecules, forming a complex network of neuron-glial connections in health and sickness. Emerging data reveals that metabolic changes in glial cells can cause morphological and functional changes in conjunction with neuronal dysfunction under disease situations, highlighting the importance of neuron-glia interactions in the pathophysiology of neurological illnesses. In this context, it is required to improve our understanding of disease mechanisms and create potential novel therapeutics. According to research, synaptic malfunction is one of the features of various mental diseases, and glial cells are acting as key ingredients not only in synapse formation, growth, and plasticity but also in neuroinflammation and synaptic homeostasis which creates critical physiological capacity in the focused sensory system. The goal of this review article is to elaborate state-of-the-art information on a few glial cell types situated in the central nervous system (CNS) and highlight their role in the onset and progression of neurological disorders.

## 1. Introduction

**Figure 1:**
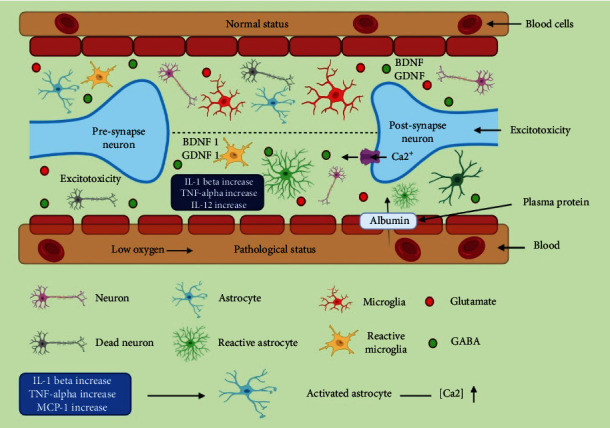
Changes that are caused by interactions between neurons and glia during neurodevelopment. Increased levels of the inflammatory cytokines IL-12, IL-1, and TNF-*α* are associated with neurodevelopmental disorders. The resulting astrocyte and microglia cell proliferation leads to their enlargement, which causes more of the molecules to be released into the extracellular space, leading to neuronal death. Albumin levels increase in patients with epilepsy due to increased permeability of the blood-brain barrier (BBB) and activation of astrocytes. In epilepsy, released cytokines have been demonstrated to promote effective neurogenesis and the synthesis of neurotrophins such as BDNF, NGF, and GDNF (NGF, BDNF, and GDNF).

Kettenmann and Verkhratsky used the term “glia” from the Greek word “stick” to describe the filling of gaps between neurons in the focal sensory system in 1856. Despite the relentlessness of neuron-focused exploration for a long period of time, Kettenmann and Verkhratsky successfully noted the significance of glial cells in understanding the role of the central nervous system in his 1858 address: “Up to this point, courteous fellows, in having thought about the sense organs, I have discussed the deeply anxious pieces of it. But, if we need to examine the sensory system in its standard-setting in the body, we should initially comprehend the substance between the legitimate neural components, hold them together, and give the whole its shape somewhat” [1]. The intricacy of the collaboration among neurons and glial cells is simply starting to be seen today. Improved coculture strategies have helped in the exploration of a few issues more noteworthy profundity. In the CNS, three glial cells (astrocytes, oligodendrocytes, and microglia) are generally perceived, each with individual capacities [2]. All four cell types have been linked to the development of essentially all known CNS pathologic conditions, including neurodegenerative diseases like Alzheimer's disease (AD), Huntington's disease (HD), Parkinson's disease (PD), amyotrophic lateral sclerosis (ALS), spinal muscular atrophy (SMA), multiple sclerosis (MS), and various pathologies like Rett syndrome [3, 4]. Accordingly, powerful model frameworks for unwinding the exceptional job of every particular glial cell type in an infection state, just as contemplating their dynamic interaction, might be incredibly valuable in recognizing novel therapeutics [5]. The first few years of life are crucial for neurodevelopment. Synapse formation and pruning, as well as the consolidation of neural circuitry, are all part of maturation [6]. The creation and pruning of synapses have traditionally been researched in conjunction with the formation and removal of synapses. Synapses were considered to operate as a messenger among two adjusting neurons, and pruning synapses might cause synapse patterns that influenced neuronal communication [7, 8]. Previously, the role of glial cells was unknown, but recent research has revealed few information such as the generation of gliotransmitters and cytokines, which allow them to interact with neurons during brain development. Moreover, neuron-glia interactions are being studied more and more in terms of synaptic alterations made by glial cells at various stages of development, like synaptic patterning as a consequence of pruning [9]. Glial cells secrete gliotransmitters such as glutamate, gamma-aminobutyric acid (GABA), and cytokines, which have direct and indirect effects on neurons [10]. These drugs have been found to affect tripartite synapses. Tonic gliotransmitter release, which is usually visible as extracellular glutamate and GABA, is also another key part of neuron-glia interactions. Tonic inhibition has been discovered to be particularly useful in the research of neurodegenerative diseases including AD and PD [11]. The chronic stimulation of nerve cells, on the other hand, has only been studied sporadically in neurodevelopmental diseases [12, 13]. For proper brain function during development and adulthood, the connections between neurons and glia are essential. According to recent studies, glia play a crucial role in bidirectional communication with neurons, adaptation to various diseases, modulation of neuronal activity, and phenotypic changes in response to neuronal injury [14, 15]. Neurons are strongly connected with glial cells such as astrocytes, oligodendrocytes, and microglia throughout the brain tissue, and their dynamic interactions are critical for appropriate brain function [5, 16]. Every type of neurological disease is thought to include a glial component, which could be the primary or secondary cause [17]. As a result, glia's protective and homeostatic abilities define their central position in neuropathology. The mechanisms governing glial cells' varied reactive states are yet unknown; however, they can be linked to changes in their metabolic profiles. In the context of these phenotypic changes in neurological illnesses, the unique metabolic changes combined with mitochondrial modifications in activated glial cells are receiving attention [5, 13, 18]. Changes in glial cells' metabolic profiles have been found to disrupt neuron-glial and interglial interactions, increasing the ongoing reaction to the initial cause. In numerous neurological illnesses, abnormal neuron-glial interactions have been identified in several studies. The rising body of evidence demonstrates the close relationship between glia and neuronal cells, as well as their impact on neurons. Several studies have found that metabolic changes in glial cells modify neuron-glial connections, hence amplifying the pathophysiology of many neurological illnesses [19–23]. In preliminary research on neurodegenerative, ischemic brain injury, and demyelinating illnesses, glial metabolism was found to have a lower oxidative burden, lower generation of proinflammatory cytokines, and less neuronal damage. As a result, future research into the underlying processes that regulate metabolic changes in reactive glial cells will lead the way for the creation of new therapeutic approaches [24–26]. In this review work, we looked into neuron-glia interactions in the setting of a number of neurodevelopmental disorders, including autism spectrum disorder (ASD), attention deficit hyperactivity disorder (ADHD), and epilepsy (Figure 1). Besides from that, many neurotransmitters and their actions have been discussed, as well as the significance of glial cells in neurological disorders.

## 2. Neurotransmitters

### 2.1. Endocannabinoids

Endocannabinoids (ECBs) are retrograde-acting molecules that are delivered from neurons in light of depolarization-initiated Ca^2+^ inundation. Depolarization-induced suppression of inhibition (DSI)/excitation flagging gave the evident primary proof to retrograde ECB flagging depolarization-induced suppression of excitation (DSE). Later examination uncovered that the ECB framework is involved in both excitatory and inhibitory neural connections as well as present moment and long-term depression (LTD). From that point forward, the ECB framework has become the mind's most investigated retrograde flagging framework. Much of the time, ECB-interceded retrograde flagging starts with the union of 2-arachidonoylglycerol (2-AG), which is set off by raised intracellular Ca^2+^ fixation and initiated Gq/11-coupled receptors. 2-AG is in this way delivered into the extracellular space, where it goes until it comes to the presynaptic terminal, where it ties to the cannabinoid receptor type 1 (CB1R). Initiated CB1R hinders synapse discharge as follows: first, by obstructing voltage-gated Ca^2+^ channels, which limit presynaptic Ca^2+^ deluge, and second, by restraining adenylyl cyclase (AC) and the cAMP/PKA pathway, which is associated with LTD [27, 28].

### 2.2. Glutamate

Glutamate is the essential excitatory synapse in the focal sensory system, which deals with astrocytes in an assortment of cerebrum regions. Despite the fact that a few studies have shown that astrocytes have ionotropic glutamate receptors, it is commonly assumed that glutamatergic communication in such glial cells occurs mostly through metabotropic glutamate receptors (mGluR). Based on their organization homology, G-protein coupling, and ligand selectivity, mGluRs are categorized into three classes. Group I contains mGluRs 1 and 5, group II contains mGluRs 2 and 3, and group III contains mGluRs 4, 6, 7, and 8. The measures of mGlu1, mGlu3, and mGlu5 receptors in astrocytes fluctuate contingent upon the formative stage and cerebrum area. According to a large research collection, mGluR5 is the most important glutamate receptor transmitted in astrocytes in culture and in situ. Ca^2+^ waves in astrocytes are triggered by glutamatergic neuronal afferent fibers in hippocampal slices, which are blocked by mGluR5 antagonists. mGluR5s are additionally significant in astrocytic reactions to glutamatergic neurotransmission in other brain regions like the core accumbens and the thalamus. Moreover, astrocyte Ca^2+^ reactions are defenseless to mGluR5 opponents when tangible incitement is acted in vivo. The statement of mGlu5 in astrocytes is high during pregnancy and reduces during advancement, when mGluR3 is upregulated, recommending that mGluR5 may just play an unobtrusive capacity in grown-up stages [29, 30].

### 2.3. Acetylcholine

Brown was the first to identify acetylcholine (ACh) as a neurotransmitter based on its effects on the heart [31]. ACh's many roles in synaptic communication have been discovered [32]. Cholinergic circuit disruption is assumed to be at least partly responsible for the cognitive impairments seen in neurodegenerative illnesses. Cholinergic circuit disruption has been connected to both normal and abnormal cognitive performance [33, 34]. While direct cholinergic activation of pre- and postsynaptic neuronal receptors is thought to be the method by which cholinergic signaling modulates cognitive functions, the involvement of ACh in astrocytes has been overlooked. Muscarinic and nicotinic receptors have been found in astrocytes [35–38]. In hippocampal slices, cholinergic agonists or synaptically produced ACh causes astrocyte Ca^2+^ increases. Such effects are mediated via muscarinic receptors, according to pharmacological techniques [39–41].

### 2.4. Norepinephrine

The neural connection norepinephrine is essentially conveyed by the locus coeruleus (LC) and has a vast extent of effects across the frontal cortex. Norepinephrine, as various catecholamines, is transmitted through axonal varicosities in neurons in this way acts through “volume transmission.” Astrocytes have adrenergic receptors a1, a2, and ß1 [42, 43] and can respond to norepinephrine made by neurons [44, 45]. *In vivo*, LC neuron impelling causes short statures in cortical astrocytic Ca^2+^, controlled by phentolamine, an ambiguous adrenergic receptor blocker [44]. Plus, advancement authorizes astrocyte networks across many psyche areas, curbed by adrenoceptor adversaries [46, 47].

### 2.5. Dopamine

Although the transport of dopamine in cultured astrocytes has been studied in great depth, the existence of down-to-earth dopamine receptors in situ has stayed a question mark until continuous assessments revealed astrocyte response to dopamine in a variety of brain locations. Dopamine D2R order of astrocytes has been demonstrated to suppress a crystallin-mediated neuroinflammation *in vivo* [48] and lower intracellular Ca^2+^ levels in hippocampal [49] and ventral midbrain astrocytes. In contrast, exogenous dopamine inception of D1R lifts intracellular Ca^2+^ in hippocampal astrocytes [49]. Moreover, we have displayed the presence of D1Rs in astrocytes of the center accumbens using electron microscopy. That inception of these receptors *in vivo* and situ by synaptically conveyed dopamine causes intracellular Ca^2+^ ascends through a GPCR hailing course, including IP3R2 and intracellular Ca^2+^ arrangement [50].

### 2.6. GABAA Receptors

GABAARs are integral membrane ion channels with five sections that govern the most common type of fast regulatory neurotransmission in the brain [51, 52]. They are sensitive to Cl and HCO3 anions. There should be at least 19 different GABAAR subunits genes, including 6*α* (*α*1-*α*6), 3*β* (*β*1-*β*3), 3*γ* (*γ*1-*γ*3), 3*ρ* (*ρ*1-*ρ*3), and also 1 gene for each of the subunits [53]. As a consequence of this diversity, various homomeric or heteromeric subunit mixtures exist, each with its location in the CNS and functional and pharmacology features [54]. A few elements impact the subunit profile that creates GABAARs, for example, cerebrum area, cell type, formative stage, and physiological or neurotic conditions [55–57]. As of late, 11 GABAAR subtypes with various subunit arrangements have likewise been found, most of which are heteromeric receptors shaped by *α*x*β*x*γ*x or *α*x*β*x*δ*, while some are homomeric receptors comprising of subunits [58, 59].

### 2.7. GABAB Receptors

GABABRs are slow and long-acting G-protein-coupled receptors (GPCRs) that are occupied with GABA-intervened restraining transmission. It was first distinguished pharmacologically as bicuculline-harsh metabotropic receptors that were animated by the GABA simple baclofen [60]. GABABRs are heterodimers made out of GABAB1 and GABAB2 receptor subunits that act together to initiate signals [61, 62]. GABAB1 has ligand binding sites, while GABAB2 has allosteric modulator binding sites [63, 64]. It is needed to get the heterodimer to the cell membrane, where the receptors can activate [65, 66]. It has an interaction with the Gi/o protein. Voltage-gated Ca^2+^channels (VGCC), inwardly rectifying potassium channels (Kir), and adenylyl cyclase are some of the effector components involved with GABABR flagging pathways in neurons [67]. However, depending on the cell type and location studied, the specific coupling of GABABRs to the molecular effector can vary [68].

### 2.8. Serotonin

Serotonergic neurotransmission is suspected to be involved in several mental diseases [69]. Even though the evidence appears to be conflicting, the role of serotonin in learning and memory has attracted interest [70]. Furthermore, experimental evidence suggests that stimulating serotonergic neurotransmission reduces behavioral performance, while inhibiting it, improves it. 5-HT 3 antagonists [71], which were shown to improve rodent and primate performance in a variety of cognitive tests [72], have yielded promising results. As a result, it is not unexpected that many substances have been created to treat AD (for example, ICS 205930, Ondansetron, and Zacopride; see [73]). A putative neurochemical mechanism of action has also been proposed by many studies. 5-HT 3 receptors appear to regulate cortical ACh release and may work through another 5-HT receptor subtype [71]. The 5-HT 3 antagonists' apparent cognition-enhancing benefits are hypothesized to be amplified by their effects on ACh generation in the brain. Because the entorhinal cortex has a higher density of 5-HT1A receptors [74], and this receptor subtype is involved in learning and memory, it has been hypothesized that it could be a target for cognitive-enhancing drugs. Several research looked into the involvement of the 5-HT1A receptor subtype in learning and memory; however, most found no evidence of improved learning or memory after using 5-HTIA agonists [75–77]. In actuality, there is no influence or impairment in learning performance. Only one study [78, 79] discovered that ipsapirone, a partial agonist of the 5-HTjA receptor, improved performance in a conditional delayed discriminating task. It will be fascinating to see if a 5-HT1A antagonist will improve both memory and learning.

### 2.9. Excitatory Amino Acids

Glutamate, the most prevalent endogenous excitatory amino acid in the brain, has attracted a lot of attention because of its possible role in neurological and psychiatric disorders [80]. The importance of NMDA and AMPA receptors in long-term potentiation indicates a link between excitatory amino acids and learning and memory activities. The physiological correlate of memory formation has been considered to be long-term potentiation [81]. Although there is evidence that blocking the NMDA receptor can affect both long-term potentiation and memory, increased glutamatergic signaling may have detrimental repercussions for behavior since excessive levels of glutamate are neurotoxic [78]. As a consequence, excitatory amino acid receptor agonists' memory-enhancing effects may be limited. However, a newly developed pharmaceutical (i.e., 1-(1,3-benzodioxol-5-ylcarbonyl)piperidine), which was expected to boost AMPA receptor activity, was found to improve cognition in different learning and memory models [79]. Finally, there seems to be a glutamatergic shortage in AD [82].

## 3. BBB Structure

**Figure 2:**
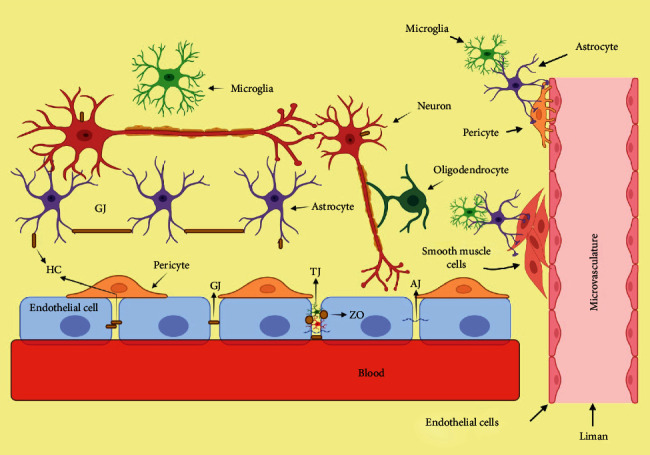
Connexin-based channels in the blood-brain barrier. Capillary endothelial cells (ECs), pericytes, neurons, glial cells, and microvasculature involvement make up a functional unit.

An essential constituent structure in the blood-brain barrier (BBB) is glial cells. Pericytes and endothelial cells (ECs) collaborate to form a continuous, membrane network around blood vessels that allows for molecular signaling (Figure 2). The barriers have strong selectivity for necessary nutrients, which prevents hazardous materials from entering the brain and keeps brain homeostasis stable. BBB thus serves a vital role in keeping the unique neuronal function in the systemic circulation safe from biochemical attack.

## 4. Glia's Role in a Healthy CNS

In the CNS, three kinds of glial cells (astrocytes, oligodendrocytes, and microglia) are traditionally differentiated, each with specific roles. Due to their diverse functions, polydendrocytes or NG2(+) oligodendrocyte precursor cells (OPCs) could be respected as a fourth glial cell type [80]. Practically completely known CNS pathologic conditions, including neurodegenerative problems like AD, PD, ALS, HD, MS, and SCI, are influenced by each of the four cell types [83–91]. Therefore, reasonable model frameworks for explaining the unmistakable jobs of each glial cell type in a sickness state and looking at their dynamic interchange could be gigantically significant in the advancement of new therapies. In the CNS, astrocytes are the most common cell type. As the nervous system becomes more complicated, their ratio and amount to neurons increases, demonstrating their importance in the development and maintenance of this complex system [92, 93]. The astrocyte population is exceptionally diverse in shape and gene expression, which aligns with the numerous roles of this cell type [94–96]. The fundamental job of astrocytes in the CNS is to maintain and provide homeostasis. Ion, neurotransmitter and neuro-hormone trafficking, metabolic support for storing and dispersing energy substrates like lactate, cellular homeostasis (neurogenesis), and organ homeostasis for constructing and maintaining the blood-brain barrier (BBB) are all examples of this [91]. Additionally, astrocytes integrate and coordinate synaptic and nonsynaptic impulses, as well as impact neighboring cell activity in a flexible manner [97, 98]. Initially, astrocytes were thought to overlap, but new data reveals that they are structured systematically, with individual cells covering separate territories and interacting with both the microvasculature and neurons. They create a tripartite synapse with neuronal transmission, and activation is modulated by pre- and postsynaptic neurons. A single astrocyte may touch hundreds of synapses simultaneously due to its numerous processes and branches [99]. Furthermore, astrocytes are linked by gap junctions that connect neurons to form a complex network that sends messages via Ca^2+^ waves at a much slower rate than neuronal communication [100].

### 4.1. Microglia

**Figure 3:**
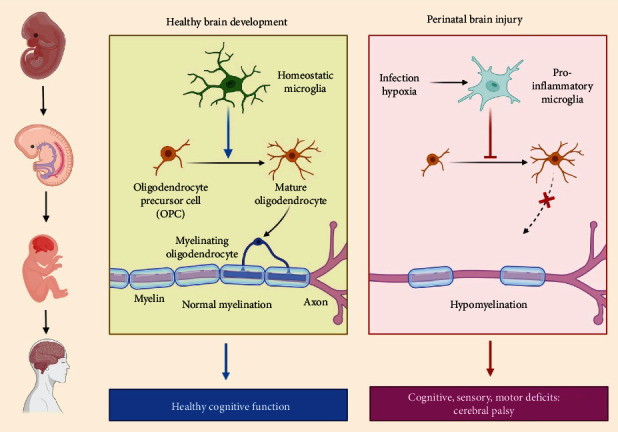
Differential roles of microglia in the developing brain. By phagocytosing dead or dying cells, microglia can control the amount of neurons in the growing brain and give neural progenitor cells trophic support for growth and maturation.

Microglia are CNS tissue-explicit macrophages with an extended life expectancy that make around 15–20 percent of the synapses. They come from the yolk sac's mesodermal hematopoietic undifferentiated cells, in contrast to the ectodermal produced neurons, astrocytes, and oligodendrocytes. Microglia antecedents (myeloid forebear cells) arrive at the CNS during early-stage improvement before the BBB is made [101]. As the name says, microglia are a lot more modest than astrocytes. They exist in an amoeboid transitory state when they enter the CNS or are set off and a ramified “resting” shape with a minuscule soma and broad praiseworthy cycles when they are not enacted. They are equally scattered all through the grown-up CNS, with every cell having its particular area (like astrocytes). Because of their immobility and lack of activation markers, “resting” microglia were thought to be dormant until recent research revealed that their fine ramified processes are constantly monitoring the environment [102]. Microglia (Figure 3) go about as immunological assessors in the sound CNS and are fundamentally liable for eliminating waste. To consistently pass on their amazing well-being to the microglia, neurotransmitters and neurotrophins are delivered by neurons and astrocytes [92, 93, 103–105]. Microglia, similar to neurons, have an assortment of synapse receptors that distinguish neuronal action and direct microglia movement, provocative reactions, cytokine delivery, neuroprotection, and neurotoxicity [106, 107]. Microglia are insusceptible cells with chemokine, cytokine, and supplement factor receptors that produce modulatory substances like cytokines and responsive oxygen species (ROS). Antigens are conveyed to attacking T lymphocytes through the significant histocompatibility complex (MHC) class II complex. After detecting a physical issue or obsessive affront, microglial cells quickly change into an amoeboid shape and move towards the site of the sore [108–110]. Microglia cells relocate towards injured or dead neurons because of glutamate-initiated Ca^2+^ waves, as per new exploration [111].

### 4.2. Oligodendrocytes

Myelination of neuronal axons is carried out by oligodendrocytes in the CNS, which is required for rapid electrical signal transmission. OPCs arise in many brain areas throughout development and travel great distances to reach their eventual destination. OPCs go through complicated proliferation and differentiation processes throughout this process. Myelination begins immediately after birth, once the OPCs have finished their migration to their site of activity. In humans, the majority of myelination occurs within the first year of life; it continues in some areas of the CNS until young adulthood [112] and throughout adulthood [113]. The development of cognitive ability in specific regions appears to be linked to myelination in those areas [97, 98, 112]. When immature oligodendrocytes come into touch with target axons, they undergo a complicated process of differentiation that entails wrapping their plasma membranes around the neurons [108, 114]. The cytoplasm is drained as the membrane layers thicken, and the residual sheets contain up to 160 compact membrane layers of myelin lipids and proteins [109, 115]. The myelination process and the development of OPCs into adult oligodendrocytes are carefully controlled. Due to a lacking of model systems, the signaling pathways and substances involved are currently unknown [116, 117]. During their development from OPCs, oligodendrocytes appear to only myelinate for a brief period [118]. While electrical driving forces in neurons are essential for myelination to start, astrocytes assume a part in the wrapping's effectiveness and speed. Oligodendrocytes have a layer that can uphold multiple times the heaviness of their cell body. An oligodendrocyte can create up to 5000 m^2^ of new layer each day during top myelination, which is a huge metabolic exertion that requires a ton of oxygen and adenosine triphosphate (ATP), just as a ton of endoplasmic reticulum limit [106, 115, 116]. Despite the way that these phones live for quite a while in a solid neurological framework, with a turnover pace of just a single cell in 300 every year, they are helpless against injury and stressors such aggravation and oxygen hardship [107]. The leftover NG2(+) OPCs scattered all through the grown-up CNS can supplant lost oligodendrocytes (for additional data on NG2(+) cells, see the part underneath). Oligodendrocytes give trophic variables to neurons and control axon width and particle direct dissemination as well as giving protection [117]. Hole intersections (like those found between astrocytes) interface oligodendrocytes with astrocytes, taking into consideration the dispersion of particles and little atoms, metabolic trade, spatial buffering, and electrical coupling [119]. To get signals, for example, certain MHC subtypes, supplement factors, cytokines, chemokines, glutamate receptors, and cost-like receptors, oligodendrocytes produce and express invulnerable administrative synthetic compounds and receptors [110]. This shows that oligodendrocytes play a part in irritation and are firmly connected to microglia.

## 5. Extracellular Vesicles as Neuron-Glia Communication Mediators

Glia cells assume a basic part in the creation, upkeep, and capacity of the focal sensory system, all of which requires critical cell-cell associations among glia and neurons. Direct cell-cell association or the paracrine activity of delivered atoms can help intercellular correspondence. A novel contact method based on cell-to-cell exchange of extracellular vesicles (EVs) has emerged in recent years. Numerous cell types discharge EVs into the climate, which can move an assortment of biomolecules between cells over brief distances or longer distances. EVs are discharged by both glia and neurons, and new examination shows that EV intercellular correspondence in the CNS has an assortment of practical implications [91, 116, 119, 120]. Size, payload, layer piece, and beginning of extracellular vesicles, for example, shedding microvesicles (MVs), exosomes, and apoptotic bodies, shift. Apoptotic bodies are delivered during apoptosis, while sound cells make various types of vesicles. EVs can be detected in practically all bodily fluids, and distinguishing between them has proven difficult due to several classification criteria overlapping [121]. In contrast to MVs, which are generated directly from the plasma membrane and range in size from 50 to 100 nm, exosomes are formed through the endosomal system (up to 1000 nm in diameter). Exosomes are intraluminal vesicles of multivesicular bodies (MVBs); hence, the ESCRT (endosomal sorting complex required for transport) process is necessary to sort them at the endosomal limiting membrane [80, 110] or aided by ceramide and tetraspanins, two sphingolipids [89, 122]. Exosomes are framed when MVBs combine with the plasma film and Rab GTPases; for example, this cycle is controlled by Rab27 in epithelial cells and Rab35 in oligodendrocytes [102, 108]. Tetraspanins, integrins, heat shock proteins, biogenesis-related proteins (such as Tsg101 and Alix), and components specific to different cell types can all be present in exosomes. However, other intracellular structures such as the mitochondria and endoplasmic reticulum are not included [92, 123]. The composition and biogenesis of MVs are less well understood. Exosome-forming components may, interestingly, be necessary for MV production by the molecular machinery [97, 113].

## 6. Neuronal Doctrine Challenged: Glial Cell Shape Brain

Only a million years ago, the quick growth of thinking, and thus of humanity, remained the major puzzle in our understanding of ourselves. The sudden advent of intelligence, and thus an only around a million years ago, human beings appeared. The critical question for our self-understanding has not been solved yet. In the same way, we have no idea how the human mind is more developed than animals. And actual variation lies between animal and human being. Since the turn of the twentieth century, this neuronal doctrine has governed current neuroscience [124, 125]; the neuron is a fundamental data processing unit made up of neurons in the brain that transfer messages unidirectionally from receiving dendrites to the integrating cell. The axon's terminal branches connect the body to the axon's terminal branches. The substrate of our intellect is widely thought to be a neuronal network connected by synaptic connections. The diameter and length of neuron cells increase according to the size of the brain (mammal). Surprisingly, the structure and physiology of neurons in humans and animals are essentially comparable, as is the quantity of neurons in humans and animals. The number of synapses in rodents and human brains is approximately 1100–1300 million per mm that is more or less stable [126]. Human protoplasmic astroglial cells, the most common glia form in grey matter, have the following linear dimensions. They are about 2.75 times more significant, and their density is about 27 times higher than in a mouse brain for the same cells. Moreover, the protoplasmic astrocytes of humans have roughly 40 major processes, with far more sophisticated branching than mouse astrocytes (they have only 3–4 main functions) [127]. In contrast to all of these quantitative alterations, the CNS of Homo sapiens and other primates acquired distinct forms of astroglia, such as interlaminar astrocytes and astrocytes. Polarized astrocytes [127, 128] are not found in the brains of different species. Glial cells play a significant role in brain development. Astroglia are neuronal-glial-vascular units that compose the CNS's well-being and provide all lines of protection.

## 7. Glial Networks of Neurons (Integral Gear of Brain Activity)

**Figure 4:**
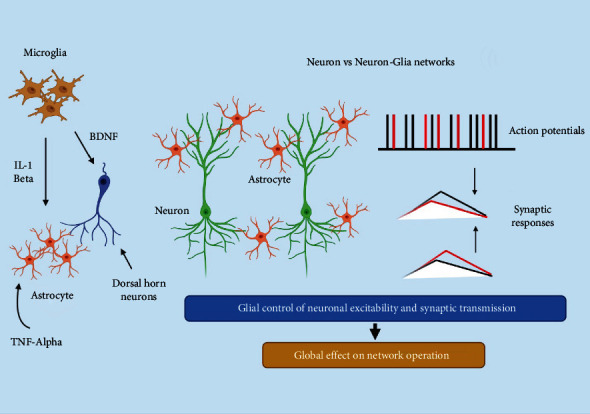
A representation of the neuron-glia network. The (red) astrocytes interact with the neurons (gray) and influence cellular excitability (top-left panel) and synaptic responses (bottom-right panel), affecting neural network function. On the right, the black and red markers show neuron-glia activity.

In the brain, information processing is usually drawn from neural activity, with neurons and their dynamic signaling pathway responsible for data transit and processing [129]. Despite this, great progress has been made in understanding the molecular and physiological characteristics of astrocytes, a kind of glial cell revealed to play a role in neurotransmission and neuronal function [130, 131]. Moreover, the active astrocyte participation in synaptic transmission, usually as neuroactive name particles released by astrocytes, describes productive signal transduction among both astrocytes and neurons [132]. There is a broad consensus that astrocytes show a critical function in the case of maintaining homeostasis of surrounding synapses, including crucial involvement in energy metabolite supply [133, 134] and clearance of extracellular potassium [135–137]. Furthermore, astrocytes that surround synapses control the extracellular space volume, as well as the amounts and flow of neuroactive chemicals outside the cell [138]. Aside from their homeostatic functions, astrocytes dynamically connect with neurons and synapses. To monitor neuronal and synaptic action, ion channels, neurotransmitter transporters, and receptors are engaged. These are activated. Molecular interactions may result in complex Ca^2+^ signals being sent to astrocytes. Astrocytes, for addition, can modify surrounding pre- and postsynaptic neuronal elements, generating functional but also morphological changes; gliotransmitters such as glutamate, ATP, and D-serine are consumed or released in the brain [139]. Synapses are the connections between neurons. Whether astrocytes are active constituents, however, remains to be shown in how neural networks work and whether or not they play a role in dynamic functions in information processing in the brain (Figure 4) [140–142].

## 8. Glial Cells' Importance in Neurological Disorders

Glial cells had originally thought to offer primarily structural and trophic aid for neurons, gluing them together (glia seems to be the Greek word for “sticky,” a phrase for glue) and providing them with essential nutrients for life. Only neurons were given the responsibility of conveying and data processing. As a result of this idea, drama has changed [143].

### 8.1. Acute Insults to the CNS

#### 8.1.1. Toxins

Astrocytes are predominantly targeted by heavy metals, which cause substantial brain damage and cognitive deficits. Because heavy metals (such as manganese, Pb, Al, and Hg) are predominantly segregated into astrocytes by diverse mechanisms, this is the case. Plasmalemmal carriers are a type of transporter found in plasma cells. Heavy metals, on average, reduce astroglial transcription of carriers of glutamate, resulting in a reduction in glutamate discharge and neuroinflammation [144–147]. Minamata illness is the name given to methylmercury poisoning, which was initially identified in the Japanese city of Minamata [148]. Ocular defects, peripheral deficits, central defect, deafness, weakness, and convulsion are signs of Minamata disease. Methylmercury is mainly found in astrocytes where glutamate and cystine uptake is inhibited [149].

#### 8.1.2. Neurotrauma

The traumatic brain and neurological disorders are categorized per their origin (penetrating wounds or concussions; it is referred to medically as cervical cord neurapraxia when it occurs in the cervical spinal cord), and severity like it can be more harmful or less. It can influence life risk, physical disabilities, mild impairment, healing location, and anatomical location [150]. A severe event to the central nervous system, by its character, has complicated pathogenesis connected mostly with direct damage to neural cells and the whole system, including loss of the brain stem capillaries and the BBB. Neurotrauma primarily induces an astrogliosis response, reaching highly reliant on the pathogenic environment [151–155]. But in the aftermath, a neurotrauma astroglial scar border develops, identifying and segregating all sites of the focused lesion from the brain, which is sound. Prevention during astrogliosis, resulting in a distorted astroglial defect, worsens cellular injury and the neurologic deficiency [156–161].

#### 8.1.3. Stroke

Astrocytes assist neurons in the case of ischemic penumbra via several homeostatic mechanisms. Importantly, the astrocyte regulates the balance of glutamate in the ischemic area of the brain. They also provide metabolism resources like lactate to neurons. In the situation of ischemia, the neuroprotective effect is increased by lactate [162]. Glutamate inflammation, which invariably occurs after a heart attack, is nearly entirely the responsibility of astroglial units. The infarct size is increased when the astroglial glutamate channel GLT-1 is found [163].

### 8.2. Epilepsy

A gradual depolarization in neurons is known as paroxysmal depolarization shift, and it is the cellular substrate of epilepsy and coincides with all cells inside an epileptic focus. Ionotropic glutamate receptors were engaged to release glutamate continuously in the multiple surrounding neurons in epileptic focus, which are responsible for such a depolarization. Epilepsy is linked to a large amount of reactive astrogliosis and the formation of a glial scar. Reactive astrogliosis germinates itself in the initial phases of these diseases, even before the clinical presentation of seizures. In epileptic tissue, the reactive astrocytes lose their domain structure. Such trait has been detected in human postmortem samples and animal models [164]. Ionotropic and metabotropic glutamate receptors are overexpressed in astrocytes from epileptic tissues, while inwardly rectifying K^+^ channels and aquaporins are underexpressed. Additionally, epilepsy decreases glutamine synthetase and astroglial plasma membrane glutamate transporter expression and function, resulting in abnormal glutamate and GABA homeostasis [137, 165–169]. Epilepsy has been linked to changes in astrocytic intracellular and intercellular Ca^2+^ dynamics [170].

### 8.3. Alexander Disease

Alexander disease (AxD) is a neurodegenerative disease that is rare, chronic, and generally fatal. It is named after William Stewart Alexander, a neuropathologist who first identified it. In pathophysiological terms, AxD is a leukodystrophy with a distinct phenotype and a primary hereditary astrogliopathology [171]. A significant gain of function variation in the GFAP gene causes AxD. This causes astrological pathology which causes significant harm to the increasing white matter. AxD is characterized by the development of protein aggregates known as Rosenthal fibers around astroglial nuclei and endfeet [171]. Megalencephaly, seizures, spasticity, speech issues, and swallowing are just a few examples of the severe mental and physical difficulties that are present in type I AxD. Type II AxD has a later onset and slightly different and less severe clinical manifestations, such as ataxia, visual and motor difficulties, autonomic dysregulation, sleep disturbances, hyperreflexia, and problems speaking and swallowing [172].

### 8.4. Neurodegenerative Disorders

As recently said, neurodegenerative infections, for example, AD, PD, and HD, are brought about by an assortment of pathologies with an assortment of hidden causes. Therefore, we have a restricted handle of the beginning phases of numerous illnesses, making it hard to recognize causes from results and hindering a total cognizance of the essential components [173]. One of the most important variables in increasing therapy outcomes is early intervention. New reprogramming and culturing approaches are fascinating tools for understanding disease development early on. Even though diverse subtypes of neurons are destroyed, they all develop distinct characteristics as the disease progresses. Because aging is a significant risk factor for many diseases, cellular care may have a significant impact on disease progression. Protein aggregation, protein trafficking, and energy metabolism disturbance, as well as oxidative stress and the production of free radicals, are all common occurrences [165, 174]. Glial cells of the CNS, which are in charge of metabolic, cellular, and transfer signals, play a big role in these systems. Receptive gliosis, which is characterized as glial cell initiation and multiplication because of injury, happens in every single neurodegenerative ailment [175]. All glial cell types are implicated in neurodegenerative diseases, and a discussion of them would be beyond the scope of this article [176–178]. It has been discovered that in ALS, engine neurons, then again, control the beginning of the illness, while astrocytes and microglia are dominatingly engaged with infection movement, suggesting that altering the reactions of these phone types could bring about significant advantages for patients [179–181].

### 8.5. The Interaction of Neurons and Glia in Alzheimer's Disease

**Figure 5:**
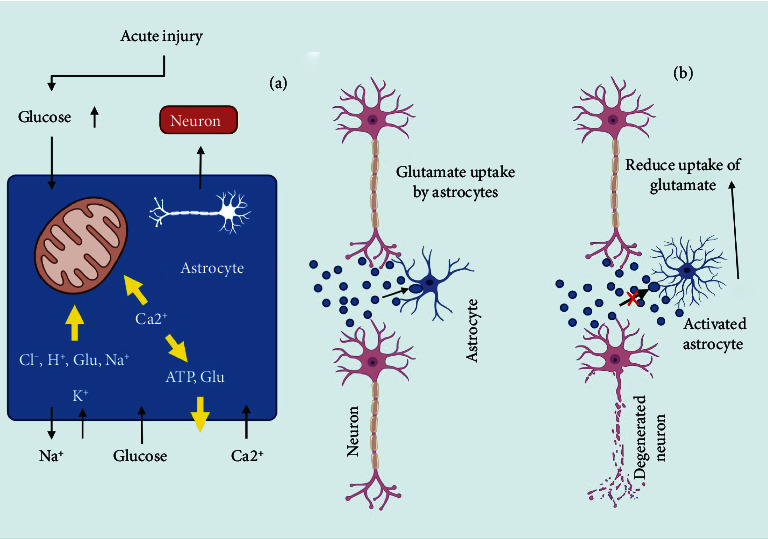
Reduced glutamate uptake by activated astrocytes.

**Figure 6:**
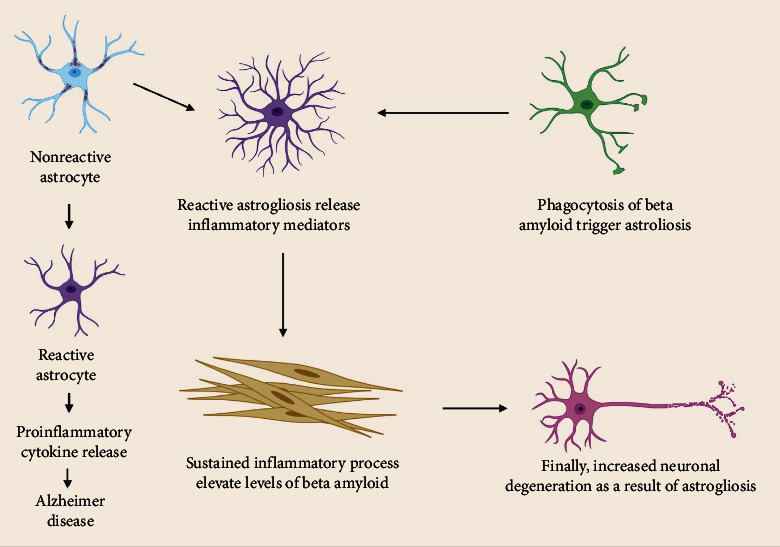
Representation of astrogliosis causing for neurodegeneration.

The majority of neurodegenerative diseases are classified as “proteinopathies,” or toxic protein clumps [182–184]. Protein aggregation most commonly happens in synapses, resulting in synaptic dysfunction [185]. Aside from proteinopathies and neuron degeneration, new research suggests that glial cells are active actors in neurodegenerative illnesses. Microglia and astrocytes are part of the network that helps the brain connect by deleting superfluous synapses [186]. Glial-based synapse elimination is reduced with age and disease. Increased complement cascade activation generates extra chemical disposition in the synapse, which disrupts the clearance process, especially in neurodegenerative conditions. Several animal models of dementia and human investigations have shown that elevated levels of C3 and C1q (components of the complement cascade) contribute to synapse loss. In AD, glutamate excitotoxicity leads to synaptic weakening due to glutaminergic signaling abnormalities [4, 187, 188]. In amyloidopathy AD models with activated microglia-mediated synaptic engulfment, C1q knockout aged mice lacking C1q-protected synaptophysin loss in the hippocampus were studied [189]. The buildup of A peptide at synaptic locations in the AD brain occurs long before the extracellular plaque aggregation that causes synaptic structural abnormalities [185, 190]. Astrocytes can change their shape and function in response to a diseased situation, which is known as astrogliosis [191]. According to De Strooper and Karran, astrogliosis is characterized by hypertrophy, multiplication, and overexpression of the glial fibrillary acidic protein (GFAP) [192]. Under pathological situations, astrocytes fail to perform their natural functions, such as maintaining K^+^ and glutamate balance, resulting in neuron depolarization (i.e., elevated Ca^2+^) [191]. Glutamate uptake by astrocytes is required for neuronal cell protection; abnormal glutamate uptake causes neuronal cell injury [193]. Several investigations have suggested that amyloid protein buildup triggers astrogliosis; nevertheless, activated astrocytes produce an A42-immunopositive substance that changes in concentration across the cerebral cortex in AD brains depending on the severity of the disease. Although the precise method by which A42 material collects inside active astrocytes is unknown, it is thought to be owing to either phagocytosis or endocytosis [194–199] (Figures 5 and 6).

Reactive astrogliosis can produce inflammatory molecular mediators that cause persistent inflammation, which is related to the development of AD [200]. Inflammatory cytokines released by microglia and astrocytes stimulate the release of secretase-1, a critical enzyme in the synthesis of amyloid protein; they also affect phagocytic activity, which is responsible for amyloid protein breakdown and clearance. Aside from the modification in the synthesis of neurotrophic mediators, another function of cytokines is the suppression of LTP, a crucial chemical for memory in the hippocampus; both processes will result in cognitive symptoms of AD [201]. The buildup of A causes upregulation of NF-*κ*B, which drives astrocytes to produce C3. Dendritic shape and network dysfunction are altered when C3 binds to the c3aR receptor (Figure 6) [192].

### 8.6. Multiple Sclerosis, Inflammation, and Injury

Multiple sclerosis (MS) is a CNS inflammatory disease with an unknown etiology that may include metabolic, genetic, and immunological factors [202]. This is one of the most prevalent CNS inflammatory disorders, and it is thought to be caused by an autoimmune reaction directed targeting myelin [203]. An abatement in oligodendrocyte number is found in a relationship with the collection of provocative cells and responsive glial cells. In creature models of MS, microglial initiation is seen before infection starts, and it is expected to assume a part in tweaking the provocative reaction [204]. Astrocytes have a significant influence on the provocative cycles in the CNS by enacting microglia, drawing leukocytes from the outskirts, altering BBB porousness, and emitting chemokines. Astrocytes assume a part in MS improvement and other CNS fiery cycles. Oligodendrocytes are powerless against irritation-initiated injury brought about by supportive incendiary synthetic compounds and nitric oxide (NO) foster anomalies and pass on during these cycles. NG2(+) OPCs might make up for the deficiency of oligodendrocytes and remyelinate axons in the beginning phases of MS, as indicated by mice models [205]. Be that as it may, NG2(+) cells are very powerless against aggravation instigated harm, and their numbers decay extensively as MS advances [206].

## 9. Neuro-Glial Coagulonome in Diseases of Peripheral Nervous System

In the PNS, Schwann cells discharge an enormous number of proteins that are all in all known as the secretome. Neurotrophic factors and extracellular matrix proteins are among the proteins produced in response to various brain impulses [207].

### 9.1. Peripheral Nerve Injury

Many studies show that coagulation factors, particularly the thrombin pathway, have a role in Schwann cell-mediated reanimation and axonal activity. The thrombin inhibitor protease nexin 1 (PN1) is secreted in the media of Schwann cells, which was discovered in the early 1990s [208]. In the peripheral nerve crush model, thrombin and prothrombin levels are elevated. Following the injury, this improvement is associated with PN1. The Schwann cells were shown to be the producer of this PN1 [209]. The participation of coagulation is indicated by a substantial elevation of factor V levels in Schwann cells following damage [210].

The activation of protease-activated receptor 1 (PAR1) could have beneficial or harmful consequences on nerve regeneration transmission. As previously stated, increasing the thrombin levels in node of Ranvier (NOR) results in a conduction block [211]. In reaction to a sciatic nerve crush injury, thrombin levels increase [212, 213]. High amounts of this substance have been related to a negative impact on nerve function. Small quantities of thrombin produce APC (activated protein C), which can stimulate PAR1 when linked in its receptor, EPCR (endothelial protein C receptor) [214]. This stimulation has a therapeutic effect on the brain [215, 216]. PAR1 stimulation boosts cultured Schwann cells' neuroprotective and neurotrophic abilities [217]. Surprisingly, the EPCR pathway activates PAR1, which reduces thrombin in vascular permeability while boosting the protective characteristics of BBB endothelial cells. Activation of sphingosine-phosphate mediates these effects [218]. The action of sphingosine-phosphate channel stimulator fingolimod on Schwann cells also supports positive PAR1 channel activation. Fingolimod, a drug used to treat MS, encourages the synthesis of proteins that stimulate neurite growth in Schwann cells, resulting in a “regenerative phenotype” [219]. Thrombin levels rose in the first hour after damage and then returned to normal state later a week. After four days of injury, the levels of EPCR were high, notably distal for injury. EPCR has also been identified in the NOR's Schwann microvilli. The PAR1 activity for the therapeutic impact is supported by the discovery of greater EPCR levels distal to the damaged location, where Schwann cells develop quickly [212]. It has also been investigated how active thrombin is produced at wounded nerve locations. TF (tissue factor) and Xa are two crucial participants in the thrombin production system in the blood coagulation pathway. Factor X is split by TF/factor VIIa complex to produce the factor Xa, which stimulates prothrombin to active thrombin. Data indicating that medication of apixaban which is a specific factor of Xa inhibitor progress the function of motor following damage adds to the clinical concern [220].

### 9.2. Guillain-Barre Syndrome

Inflammatory neuropathies are a set of disorders in which axons, myelin, or both are damaged. Because an inflammatory response influences the NG-coagulonome and can alter Schwann-axon function, it is critical to assess its position in diseases like these. The acute inflammatory neuropathies are referred to as GBS, but inflammation of polyneuropathy is common [221]. GBS patient needs mechanical ventilation in 25% of cases, and also, patient cannot be able to walk in 20% of cases after six months, and death occurs in 3-7 percent of cases. The typical mouse model for GBS is the autoimmune animal model, which shows nerve conduction velocity reduction and NOR structure degeneration. These functional and structural changes are associated with increases in thrombin phases in the sciatic nerve and a decrease in nodal PAR1 stages. The animal flow velocity and NOR structure are balanced when given thrombin inhibitors [222].

### 9.3. Chronic Inflammatory Demyelinating Polyradiculoneuropathy

CIDP (chronic inflammatory demyelinating polyradiculoneuropathy) is a peripheral nervous system (PNS) demyelinating illness that affects gradual loss of motor and sensory abilities [223]. With the exception that it is chronic and has relapsed, CIDP has a clinical course similar to GBS. The onset is gradual, and it disproportionately affects people of a given age range [224, 225]. The resistant framework assaults and obliterates the myelin sheath of the PNS, causing demyelination and axonal degeneration in fragments [226]. Histological outcomes from the CIDP show a meager myelin sheath with more limited internodes, commonly referred to as onion bulbs. The slow nerve conduction rate, which indicates conduction block, indicates demyelination [226]. Autoimmunity to neurofascin-155 (NF155) and contactin-1 (CNTN1) has recently been discovered in a large number of patients [227, 228]. NF155 is a glial paranode-expressed adhesion molecule that binds to CNTN1, a key axonal adhesion molecule [229]. CIDP symptoms appear gradually but steadily, with neurological impairments peaking after 8 weeks of disease initiation [223]. There are a number of signs and symptoms, such as tingling and numbness in the extremities, symmetrical sluggishness and paresthesia in the arms and legs, fatigue, ataxia, and limb incoordination [226]. Therapy with oral glucocorticoids usually results in a positive outcome. Plasmapheresis and intravenous immunoglobulin (IVIG) are also successful treatments [194, 223, 230–236].

### 9.4. Neuropathy Caused by Anti-Myelin-Associated Glycoprotein

IgM monoclonal gammopathy against MAG in fringe nerves causes myelin-associated glycoprotein (MAG) neuropathy [237]. MAG is a sort I transmembrane glycoprotein present in the periaxonal SC and oligodendroglial layers of myelin sheaths, where it keeps in touch and axonal capacity [238]. At the point when MAG is lost, the integrity of the myelin sheath and axonal capacity are impaired. MAG has a carb epitope with other glycoconjugates that go about as significant antigenic destinations for IgM paraproteins [237]. Chickens are administered with serum that contains IgM antagonistic to MAG paraproteins, which results in segmental demyelination and conduction obstruction [239]. Increasing mild to severe distal muscular weakness and growing sensory ataxia and frequent tremors are all symptoms of the condition. The clinical course is usually uneventful, with only minor functional decline over time. Supportive therapy, such as exercise and balance training, is employed because anti-MAG neuropathy symptoms are typically mild and initially do not interfere with the patient's daily activities. Medication should be used to address sensorimotor weakness. Only in extreme cases are steroids, intravenous immunoglobulin, and plasmapheresis used. Rituximab, a monoclonal antibody that targets the CD20 antigen on the cell surface, is efficacious [240].

### 9.5. Nerve Pathology

Demyelination caused by macrophages is the first lesion, followed by Schwann cell growth and remyelination. Furthermore, there is a varying degree of axonal degeneration, which can become severe with time but is rarely the main feature. The motor axons' ventral roots and terminal portions are the first to be impacted. Many nerve trunks have demyelination that runs the length of them. Dispersed lesions with various degrees of demyelination and remyelination, as well as axonal degeneration, result from the pattern of evolution and distribution of lesions across the peripheral nervous system [241].

### 9.6. Vasculitic Neuropathy

PNS vasculitis can occur as a component of a larger systemic vasculitis or as a separate illness (nonsystemic vasculitic neuropathy) [242–245]. Primary systemic vasculitis [246] includes Takayasu syndrome, thromboangiitis obliterans, Kawasaki disease, Churg-Strauss syndrome, Wegener granulomatosis, cryoglobulinemic vasculitis, Behçet's disease, giant cell arteritis, classical panarteritis nodosa, microscopic polyangiitis, and Henoch-Schönlein. Vasculitis of the peripheral nervous system can take the form of a single mononeuropathy, overlapping mononeuropathies, or symmetric polyneuropathies that are far apart. Peripheral neuropathy is an important clinical feature of vasculitis, and it is often the first symptom [247–251]. The expression “mononeuritis multiplex” has been authored to depict the most common and trademark kind of vasculitic neuropathy. It alludes to the imbalanced successive contribution of explicit nerves or trunks from distal to proximal [252, 253]. Neuropathy is habitually unexpected, with torment in the influenced nerve's field, showing that both engine and tactile modalities are involved. Fleeting and Takayasu arteritis are the two sorts of monster cell arteritis, albeit just transient arteritis causes fringe neuropathy. Patients might foster a few mononeuropathies, radiculopathies, plexopathies, or a wide tangible fringe neuropathy [254].

## 10. In Central Nervous System Diseases, the Neuro-Glia Coagulonome

A growing body of literature indicates that thrombin and its related route play a significant function in CNS physiology. Some thrombin's features are moderated by glia cells inside the CNS, just because they are in the PNS. The thrombin pathway has an effect on a variety of cellular processes, which can be beneficial or detrimental depending on dosage, receptor activation technique, and downstream indicating. Extrinsic sources of thrombin include inflammation events and blood-brain barrier disruption, while intrinsic sources include glial cells. The impact of thrombin on brain activity is profound, regardless of source. As previously stated, the PAR1 pathway is involved in myelin modulation and is required for nerve function [255, 256]. In PAR1 mutant mice, the direct placement of myelin proteins throughout the spinal cord was shown to be dysregulated. An *in vitro* study found that an oligodendroglia cell lacking PAR1 had higher levels of proteolipid protein and simple myelin protein, confirming the impact on myelin regulation [255]. The stimulation of PAR1 by thrombin causes a rise in cytosol calcium and mitogen-triggered protein kinases in cultivated microglia [257]. The generation of tumor necrosis factor and nitric oxide is responsible for this [258]. Thrombin activates PAR1 in astrocytes, causing structural and physiological alterations resulting in extended retract and astrogliosis [259]. As previously stated, the NG-coagulonome affects neuronal electrical activities and nerve function in physiology, implying that it may play a role in CNS disorders. Minimal thrombin values were discovered to have protective effects inside the CNS in the last decades, giving rise to thrombin preconditioning. Before ischemia, a trim level of thrombin administered directly to a rat caudate nucleus decreased ischemia injury and brain edema [260]. The dual impact of thrombin presents where the good and detrimental impacts meet at what concentration [261].

### 10.1. Epilepsy

The widespread incidence of brain damage and seizures has prompted scientists to wonder about the mechanism that links the two. Injecting thrombin directly into the brains of rats triggered motor seizures, according to a major study [262]. This was the first of many investigations exploring the complex relationship between thrombin, trauma, and epileptic activity. In the context of brain traumas and overly high levels of protein, the dangers of thrombin in the CNS have been investigated. The PAR1 pathway is implicated in epileptic activity in mice with either too much or too little PN1, indicating that the PAR1 pathway is involved [263]. Thrombin impacts neuronal plasticity in a dose-dependent manner, similar to how it affects the PNS. Low amounts activate the aPC, but high levels cause a gradual, LTP that is NMDA-dependent [264]. PAR1 is involved in both impacts. PAR1 and aPC promote LTP in hippocampal slices in the presence of a brief tetanic stimulation via a mechanism involving sphingosine-phosphate receptor 1 [265]. Thrombin changes the electrophysiological of mouse hippocampus brain slices via activating PAR1. It induces a decrease in the epileptic threshold in CA3 neurons as well as an increase in the responsiveness of CA1 neurons to afferent stimuli (which is inhibited by NMDA receptor antagonist) [266]. On the one hand, these two well-known thrombin adverse effects are associated with memory and learning impairments and, on the other, hyperexcitability and increased seizure susceptibility. Thrombin generates more spontaneous activity potentials in CA3 neurons, which is associated with higher PAR1 expression in CA3 neurons, according to whole-cell patch monitoring of pyramidal neurons in the hippocampus [267]. Seizures are triggered by a positive feedback loop of depolarization in a PAR1-dependent manner, a rupture of the blood-brain barrier, and the entry of more thrombin, according to this discovery [268]. Epileptic seizures, status epilepticus, and brain damage can all be caused by paraoxon and other organophosphates. The brains of paraoxon-treated mice contain high levels of thrombin, PAR1, and pERK, as well as enhanced electrical impulses in the CA1 and CA3 neurons of the hippocampal. A PAR1 antagonist is used to minimize excessive electrical activity [269–275]. These findings suggest that thrombin is implicated in an epileptic activity that is not triggered by trauma, raising new issues concerning the origins of thrombin in the CNS [276]. Epilepsy models with observed neuron-glia interactions are as follows [13]:

Genetic: AP- (activator protein-) 1 KO, SCN1A KO, and DBA/2 KO; Lafora disease: Malin KO, Epm2a, and Epm2b

Pharmacological: pentylenetetrazol (PTZ), pilocarpine (PA), and kainic acid (KA)

Environmental: electrical stimulation and brain injury

∗To study epilepsy, pharmacological models are commonly and widely used.

### 10.2. Traumatic Neuronal Damage (TBI)

The term “traumatic brain injury” (TBI) covers a wide range of conditions in which the brain has been subjected to external force. Although it can cause headaches and short- and long-term cognitive impairment, mild traumatic brain injury (mTBI), which is defined as a Glasgow Coma Scale of 13 to 15 following a head injury, is relatively harmless. TBI damages the blood-brain barrier either microscopically or macroscopically; it may allow plasma proteins like thrombin to touch brain tissue, as well as the synthesis and release of endogenous brain proteases. TBI animal models come in a range of shapes and sizes, each indicating a different sort of injury mechanism. Among these are fluid percussion injuries, free weight drop devices, and piercing mechanisms. After spinal cord damage, better motor outcomes, less astrogliosis, and lower levels of proinflammatory cytokines like interleukin-1 and interleukin-6 are all observed in PAR1 deletion rats. *In vitro*, thrombin stimulates the production of inflammatory cytokines by astrocytes, while astrocytes exposed to interleukin-6 upregulate PAR1 and thrombin, creating a feedback loop [277, 278]. Thrombin was thought to play a function in the development of hyperalgesia in rats after spinal cord injury. Fibrin levels rise one day after nerve root compression as an indirect indicator of thrombin activity. To prevent hyperalgesia, the mice were administered hirudin, a thrombin inhibitor, or PAR1 antagonists. Furthermore, even when there was no prior trauma, intrathecal thrombin injection generated hyperalgesia, which could be avoided by inhibiting spinal PAR1 before thrombin injection [279]. These two spinal cord investigations show the importance of the thrombin-PAR1 pathway in a variety of inflammatory processes and their clinical consequences following CNS damage, albeit they do not explain thrombin's origin. Amnesia following a mild traumatic brain injury (mTBI) is a common event that has been explored in an animal model induced by free weight loss, which is equivalent to the definition of mild TBI. According to behavioral and memory tests, brain thrombin levels rise after mTBI, and the animals become amnestic. Amnesia is caused in the same way by intraventricular injections of thrombin or a PAR1 agonist. PAR1 antagonists prevent this amnestic effect, indicating that the PAR1 pathway is implicated in the formation of amnesia after mTBI [279].

The stabilization of thrombin activity levels in the hippocampus marks the end of trauma-induced amnesia, according to a second study employing the same animal model. In the context of brain trauma, this shows a link between cognitive abnormalities and brain thrombin levels [280]. It is tempting to believe that the presence of thrombin in the CNS after an injury is due to plasma leakage caused by a blood-brain barrier rupture. Researchers looked at the kinetics of thrombin activity after damage to figure out where it comes from. In the mTBI model, two thrombin peaks were measured. The elevation was measured immediately after the insult and then adjusted an hour later. A breach of the blood-brain barrier is most likely to blame for this increase. The second peak occurred 72 hours later, followed by an increase in PAR1 and, more intriguingly, an increase in the thrombin inhibitor PN1. This late rise is very certainly due to inflammation mediated by astrocytes [281–284]. Injecting thrombin into the brain ventricle enhances seizure susceptibility 72 hours after the insult, according to the same study, when compared to animals who did not receive thrombin but did not suffer mTBI. After mTBI, an increase in PAR1 was assumed to be the reason for sensitivity [276].

### 10.3. Ischemic Injury

Thrombosis, embolism, or systemic hypoperfusion can all induce ischemic stroke. When global ischemia was induced in rats [285], prothrombin mRNA levels increased, while PN1 and PAR1 levels remained unchanged. This calls into question the role of thrombin in ischemic injury. When a brief carotid artery blockage causes *in vivo* ischemia, it increases prothrombin and factor X mRNA levels in the ischemic core as well as thrombin activity across the ischemic hemisphere, including the peri-infract areas [286]. Synaptic responses in hippocampal slices exposed to thrombin concentrations equivalent to those found in the ischemic hemisphere are changed [286]. A considerable increase in brain thrombin level over time was seen (up to 24 hours after ischemia) in an irreversible ischemic animal model, followed by a decline in PAR1 activity in the ischemic core [287]. A factor Xa inhibitor (apixaban) taken systemically reduces brain thrombin levels and diminishes infract size shortly after ischemia induction [288]. PAR1 knockout mice have less brain edema and neuronal damage, as well as fewer behavioral problems than wild-type mice [289]. In mouse mind cuts, *in vitro* ischemia brought about by intense oxygen and glucose hardship increments hippocampal thrombin presence and action while diminishing prothrombin mRNA. During oxygen and glucose hardship, *in vitro* accounts from CA1 neurons in the hippocampus demonstrated the development of ischemia LTP, which communicates the useful harm brought about by thrombin increment. Ischemic LTP is intervened by thrombin through PAR1 and is repressed when either thrombin or PAR1 is restrained. The NG-coagulonome may be a therapeutic target for ischemic stroke, according to these studies [290].

### 10.4. Neoplasms

In the United States, primary brain neoplasms occur at a rate of 29.9 per 100,000 people [291]. Glioblastoma (GBM) is a type of glial tumor that accounts for 15% of all primary CNS cancers. It is cancer that is quite aggressive, with a 10- to 12-month median overall survival rate [292, 293]. Current therapies for tumors in conveniently accessible locations include complete excision [294]. Adjuvant treatment with radiation and the alkylating medication temozolomide [295] is essential due to the tumor's infiltrative nature. Despite this combined strategy, these drugs seldom extend median survival time by more than a few months and are severely damaging to patients. GBM processes necessitate more precise treatments. Thrombin has been associated with the formation of GBM [296–298], and PAR1 expression in glioma cells has been identified. In culture, glioma cells synthesize and secrete active thrombin, which promotes proliferation and is inhibited by dabigatran, a thrombin-specific inhibitor [299]. In PAR1 knockout mice, GBM edema volume and glioma development indicators (vascular endothelial growth factor and hypoxia-inducible factor 1) are reduced [297]. The PAR1 gene (F2R) is also in the top 2% of overexpressed genes in GBM patients, according to differential analysis of its expression in human GBM patients [300]. In human GBM patients [301], increased PAR1 levels exhibit a positive correlation with TF expression and a negative correlation with tumor suppressor factors, implying that this pathway is involved in GBM pathogenesis. In a rat GBM model, thrombin activity is increased in vivo and is linked to brain edema volume. The rise in PAR1 is only seen in the tumor and does not affect the surrounding tissues [302]. SIXAC, a PAR1 proteolytic activation inhibitor that is selective and irreversible, inhibits glioma cell proliferation, invasive, and colony formation in vitro. SIXAC was demonstrated to minimize cerebral edema and extend survival in a rat GBM model when administered directly to the tumor bed [300]. For this incurable condition, the NG-coagulonome has been proposed as a therapeutic target.

## 11. ASD and ADHD

There is presently no effective treatment for ASD as it is a neurological illness that affects 1% of the population. In ASD, there is a large gender gap, with boys having a rate that is 4.3 times higher than girls [303]. For the vast majority of persons who are impacted by ASD, the cause is unclear. Social and communication difficulties, repetitive behaviors, and excessive interests are all characteristics of ASD (Table 1). Autism is linked to both inherited and environmental factors, according to a previous study. One of the environmental elements connected to maternal immunological activation is linked to ASD. Maternal viral infection, exposure to toxins, and obesity have all been linked to inflammatory and immunological system failure, which may increase the likelihood of behavioral issues in offspring [304–306]. Mothers of children with ASD, for example, have an increased chance of allergies and autoimmune illnesses than mothers of children who are developing typically [307]. IFN-*α*, IL-*α*4, and IL-*α*5 levels were shown to be greater in the midgestation serum of women expecting a child with ASD. Neuron-glia interactions are also executed in ADHD models (Table 2).

## 12. Metabolic Interaction between Neuron and Glia: The Significance of Glia-Secreted Metabolites

Glial cells provide the majority of the energy that neurons require. For brain function, the bioenergetic connection between neurons and glial cells is essential. The metabolic coupling of neurons and glia is a multistep process involving a variety of enzymes that convert biomolecules, transporters that transfer substances between the two cells, and cell surface receptors. To carry out their jobs and preserve mitochondrial integrity and membrane potential, neurons require a lot of ATP. Endothelial cells, neurons, and glia make up the neurovascular unit, which controls the delivery of nutrients and energy supplies to neurons. Endothelial cells have receptors for a wide range of metabolic substrates, including glucose transporters 1 (GLUT1), monocarboxylate transporters for ketone bodies (MCT1 and 2), and fatty acid transporters (CD36,) [318, 319]. Endothelial cells have transporters that operate as a gatekeeper for nutrients entering the brain in a concentration-dependent manner. Intercellular contact between astrocytes and endothelial cells is essential for nutrient delivery to the brain. It was recently discovered that NO produced by endothelial cells boosts astrocyte glycolytic activity [320]. Endothelial cells that come into close contact with astrocyte end foot and therefore become active components of the neurovascular unit prevent neurons from oxidative destruction, create gliotransmitters, and give energy substrates for neurons. The astrocytic-neuron lactate shuttle (ANLS), which focuses on astrocytic responses to neuronal metabolic supports, illustrates the role of neuron-glial connections in preserving brain homeostasis [321]. In addition to astrocytes, recent studies have revealed the importance of oligodendrocytes in the metabolic maintenance of neurons, particularly the axonal regions of neurons. Both astrocytes and oligodendrocytes exhibit neuronal activity, which is detected by the extracellular glutamate that neurons release. Through GLUT1, glutamate binding to the appropriate receptors on both cell types facilitates glucose absorption. Inside the cells, glucose is transformed into either lactate by glycolysis or pyruvate for oxidative phosphorylation by the mitochondria. Glycogen, which is intracellular glucose that has been stored, can also be used by astrocytes to generate energy. Lactate is produced by oligodendrocytes and astrocytes and can either be actively transported to neurons by MCT transporters or converted to pyruvate for fatty acid or ATP generation. In order to prevent neuronal excitotoxicity, astrocytes also play a crucial role in glutamate synaptic clearance. Inside astrocytes, glutamine synthase transforms glutamate into glutamine, which is subsequently transferred to neurons to produce glutamate. Glutamate, glutamine, and tricarboxylic acid (TCA) cycle metabolism are closely related in neurons, astrocytes, and oligodendrocytes [321, 322]. Krebs cycle and citric acid cycle are other names for the TCA cycle. It is the most crucial metabolic pathway for the body's energy source. TCA is the most significant central metabolic route, connecting nearly all of the individual metabolic pathways. Since neurons can generate ATP from a variety of substrates depending on different circumstances, such as fasting or hyperactivity, glial cell metabolites are essential for maintaining neuronal energy requirements. Among the energy substrates, lactate is currently acknowledged as the predominant source of ATP synthesis during hyperactivity [323]. Since MCT2 reduction in the rat hippocampus led to memory impairment, lactate—which is mostly generated by astrocytes and oligodendrocytes—is known to have a function in memory formation. Injections of glucose did not improve the memory deficits, showing the relevance of glial-derived lactate in memory processing [324]. During hyperactivity, astrocytes use a variety of strategies to regulate neuronal metabolism, in addition to supplying lactate. Reactive oxygen species, which are generated during times of information processing, cause the phospholipids that make up cell membranes' fatty acid content to peroxide. Peroxidized fatty acids generated during vigorous neuronal activity may contribute to neurodegeneration because neurons' mitochondrial capability to utilise fatty acids for ATP production is constrained and they are unable to make lipid droplets. Recent research has demonstrated that astrocytes absorb peroxided fatty acids via lipoprotein particles and produce ATP by oxidizing fatty acids to protect neurons during times of hyperactivity. Increased reactive oxygen species (ROS) formation is countered by higher detoxifying gene expression. Additionally, enhanced inhibitory interneuron activity, which regulates excitotoxicity, is upregulated in response to glutamate released from overactive neurons by astrocytic ATP [325]. During neuronal excitation, astrocytes regulate cholesterol and fatty acid production to maintain synapse integrity and transmission. In hippocampal astrocytes, sterol regulatory element binding proteins (SREBPs) are highly expressed, govern cholesterol production in astrocytes. SREBP cleavage-activating protein (SCAP) deletion in astrocytes reduced cholesterol and phospholipid secretion. In SCAP mutant mice, immature synapses increased while presynaptic proteins decreased, inadequate short- and long-term hippocampal synaptic plasticity as a result [326]. These results demonstrate that astrocytic control of neuronal activity is mostly mediated by cholesterol and fatty acid metabolism [24].

## 13. Concluding Remarks and Future Directions

The release of trophic factors and immunomodulatory substances by cellular replacement therapy is a defining moment in neuroscience, with a promising future in replacing lost cells and fostering a neuronal survival environment. Progress has been achieved in producing human iPSC lines from a variety of CNS illnesses since the discovery of iPSCs. In a recent study, iPSC-derived NPCs from a PD patient were transplanted into a monkey model, bringing iPSC research to the preclinical stage. Krencik and coworkers revealed a groundbreaking approach for generating astrocytes from iPSCs that showed functional features of glutamate absorption, synaptogenesis, and calcium wave propagation, as well as astrocyte lineage markers. This approach can now be used to generate glial cells from sick iPSC lines, laying the groundwork for future glial therapies. Other neurological disorders, such as depression, stroke, ischemia, spinal cord injury, autism, schizophrenia, and others, are being studied for glial involvement. Glial cells are a potentially attractive therapeutic target for cell replacement therapies due to ongoing efforts to recognize glial contributions to illnesses and efforts to replace them. Much about the communication between neurons and glia is still uncertain. For example, a wide controversy rages about how many of the many chemicals released by glia in response to physiological stimuli come from the release of adenosine triphosphate (ATP); other chemical messengers include glutamate and glutamine. Glial Ca^2+^ responses are generated by a diverse set of chemical messengers via a variety of pathways and hence could be linked to neuron-glial and glial-glial communication. Because Ca^2+^ is ubiquitous as a second messenger, it is even more difficult to distinguish direct from indirect effects. We know far too little about the vast range of glia variety. It is hard to discover the processes of interaction and communication between neurons and glial cells at this early period of research because of the numerous distinctions that are expected to exist between these cells. A prominent example of this phenomenon is the similarities and contrasts between cell culture and in situ neuron-glial communication. Information currently is not sufficient to reflect the full complexity of the events. Future studies on glial variety and cell-cell communication processes will lead to an improved understanding of the roles played by nonneuronal cells in neural processing. The neural connections glial cells make with neurons in several neurodevelopmental diseases were highlighted in our review. Autism, ADHD, and epilepsy are each characterized by some mechanisms that are present in ASD, ADHD, and epilepsy, such as neuroinflammation, imbalance of excitation and inhibition, and neurotransmitters. Complementary approaches using patients and animal models indicate that there is an increase in cytokines in the brain in neurodevelopmental disorders. Neurotransmitter alteration can potentially result in a neurotransmitter imbalance. An imbalance in neurotransmitter concentrations could be caused by changes in receptor and transporter expression levels, changes in released gliotransmitters, or a malfunction in uptake.

## Figures and Tables

**Table 1:** Neuron-glia interactions are executed in ASD models.

Serial no.	ASD	Model of ASD	References
1.	Genetic	PTEN mutant, MeCP2 mutant, TSC1 HT, BTBR, Scn1a HT, Shank2 KO, Shank3 KO, NLGN3R451C KI, TSC1 HT, NLXN1 KO, BTBR, MeCP2 mutant, and Scn1a HT	[308, 309].
2.	Pharmacological	VPA (valproic acid)	[310]
3.	Environmental	MIA (maternal immune activation), methyl mercury, and polyinosinic: polycytidylic acid (poly I: C)	[307, 311–314].

**Table 2:** Neuron-glia interactions are executed in ADHD models.

Serial no.	ADHD	Model of ADHD	References
1.	Genetic	nAChR (nicotinic acetylcholine receptor) *β*2-KO, DAT (dopamine transporter) mutant, NK1R-KO, SNAP25 mutant, Cdk5 KO, Git1 KO, and DAT (dopamine transporter) mutant	[315]
2.	Pharmacological	Ethanol, methyl azoxy methanol	[316, 317].
3.	Environmental	Neonatal X-rays, hypoxia, heavy metal exposure (cadmium, lead), and oncogenic environmental exposure (polychlorinated biphenyl (PCB)) are all factors that might cause cancer in children	[315]

## Data Availability

All data are available in the text.

## Abbreviations

CNS: Central nervous system
AD: Alzheimer's disease
HD: Huntington's disease
PD: Parkinson's disease
MS: Multiple sclerosis
ALS: Amyotrophic lateral sclerosis
SMA: Spinal muscular atrophy
BBB: Blood-brain barrier
NGF: Nerve growth factor
ECSs: Endocannabinoids
DSE: Depolarization-induced suppression of excitation
LTD: Long-term depression
CB1R: Cannabinoid receptor type 1
AC: Adenylyl cyclase
mGluR: Metabotropic glutamate receptors
ACh: Acetylcholine
LC: Locus coeruleus
GPCRs: G-protein-coupled receptors
AMPA: *α*-Amino-3-hydroxy-5-methyl-4-isoxazolepropionic acid
NMDA: N-Methyl-D-aspartate
OPCs: Oligodendrocyte antecedent cells
SCI: Spinal cord injury
ATP: Adenosine triphosphate
AxD: Alexander disease
GFAP: Glial fibrillary acidic protein
PNS: Peripheral nervous system
PN1: Protease nexin 1
NOR: Node of Ranvier
EPCR: Endothelial protein C receptor
CIDP: Chronic inflammatory demyelinating polyradiculoneuropathy
MAG: Myelin-associated glycoprotein
TBI: Traumatic brain injury
NLGN: Neuroligin
NLXN: Neurexin
TSC: Tuberous sclerosis
MeCP2: Methyl CpG binding protein 2
Scn1: Sodium channel protein type 1
PTEN: Phosphatase and tensin homolog
NK1: Neurokinin
SNAP: Synaptosome-associated protein
ASD: Autism spectrum disorder
ADHD: Attention-deficit/hyperactivity disorder
GJ: Gap junctions
HC: Hemichannels
AJ: Adherens junctions
TJ: Tight junctions
BDNF: Brain-derived neurotrophic factor
GDNF: Glial cell-derived neurotrophic factor
BDNF 1: Brain-derived neurotrophic factor 1
GDNF 1: Glial cell-derived neurotrophic factor 1
IL1: Interleukin-1
IL12: Interleukin-12
TNF*α*: Tumor necrosis factor *α*
MCP-1: Monocyte chemotactic protein-1
GABA: Gamma-aminobutyric acid.
